# Impact of Biotic and Abiotic Factors on *Listeria monocytogenes*, *Salmonella enterica*, and Enterohemorrhagic *Escherichia coli* in Agricultural Soil Extracts

**DOI:** 10.3390/microorganisms12071498

**Published:** 2024-07-22

**Authors:** Dimple Sharma, Autumn L. Kraft, Joshua O. Owade, Mateja Milicevic, Jiyoon Yi, Teresa M. Bergholz

**Affiliations:** 1 Department of Food Science and Human Nutrition, Michigan State University, East Lansing, MI 48824, USA; sharmad6@msu.edu (D.S.);; 2 Department of Microbiological Sciences, North Dakota State University, Fargo, ND 58105, USA; 3 Department of Biosystems and Agricultural Engineering, Michigan State University, East Lansing, MI 48824, USAyijiyoon@msu.edu (J.Y.)

**Keywords:** soil extracts, *Listeria monocytogenes*, *Salmonella enterica*, enterohemorrhagic *Escherichia coli*, native microbiome

## Abstract

Outbreaks of Enterohemorrhagic *Escherichia coli* (EHEC), *Salmonella enterica*, and *Listeria monocytogenes* linked to fresh produce consumption pose significant food safety concerns. These pathogens can contaminate pre-harvest produce through various routes, including contaminated water. Soil physicochemical properties and flooding can influence pathogen survival in soils. We investigated survival of EHEC, *S. enterica*, and *L. monocytogenes* in soil extracts designed to represent soils with stagnant water. We hypothesized pathogen survival would be influenced by soil extract nutrient levels and the presence of native microbes. A chemical analysis revealed higher levels of total nitrogen, phosphorus, and carbon in high-nutrient soil extracts compared to low-nutrient extracts. Pathogen survival was enhanced in high-nutrient, sterile soil extracts, while the presence of native microbes reduced pathogen numbers. A microbiome analysis showed greater diversity in low-nutrient soil extracts, with distinct microbial compositions between extract types. Our findings highlight the importance of soil nutrient composition and microbial dynamics in influencing pathogen behavior. Given key soil parameters, a long short-term memory model (LSTM) effectively predicted pathogen survival. Integrating these factors can aid in developing predictive models for pathogen persistence in agricultural systems. Overall, our study contributes to understanding the complex interplay in agricultural ecosystems, facilitating informed decision-making for crop production and food safety enhancement.

## 1. Introduction

Outbreaks of Enterohemorrhagic *Escherichia coli* (EHEC), *Salmonella*, and *Listeria monocytogenes* have been linked to the consumption of fresh produce [1,2,3]. These pathogens are a significant food safety concern due to the high number of illnesses and severity of disease [4]. These pathogens can be introduced onto fresh produce in the pre-harvest environment through various routes including contaminated irrigation water, application of raw manure, direct fecal deposition by wildlife, and lapses in worker hygiene [5]. Additionally, the runoff of manure from animal farms to crop fields and water sources, as well as flooding events, can contribute to pathogen spread in agricultural settings [6,7]. The Food and Drug Administration (FDA) final water rule recognizes this risk, as farmers are required to evaluate potential for flooding as sediments in these environments can serve as a harbor site for foodborne pathogens [8]. If fields are flooded, the California Leafy Greens Marketing Agreement (LGMA) [9] requires that leafy greens are not harvested within 9 m from the edge of the flooded area due to potential contamination. It also has been suggested to not use the same flooded soil for planting for up to 60 days [10]. Soils used for production of fresh produce vary in composition and pathogen survival can differ as well [11].

The differences in physiochemical properties like pH, porosity, aggregation, and cation exchange, and biological properties including the abundance and diversity of microbes in the soils affect pathogen survival. Loamy soil supported greater *Salmonella* survival compared to sandy soil [12]. Soil texture mainly affected long-term survival of *L. monocytogenes*, while short-term (<2 weeks) survival was more influenced by soil chemical properties [13]. Adverse conditions like low pH and competition for nutrients within agricultural soils have been shown to negatively affect the survival of EHEC [14]. Other than the intrinsic variation, soil management practices also alter the physicochemical properties and thus influence foodborne pathogen survival in soils. The application of soil amendments is known to influence pathogen behavior in soils, where the presence of biological soil amendments, like dairy manure and poultry litter, has been associated with increased survival of foodborne pathogens. *Salmonella* inoculated into amended soils had greater survival compared to when inoculated into unamended soil [15]. Attenuated *E. coli* O157:H7 survived better in soils amended with poultry litter compared to soils amended with horse manure or dairy manure [16]. In establishing that amended soils had elevated levels of organic carbon, phosphorus, and nitrogen, there was a positive correlation with the survival of organisms such as *E. coli* and *Salmonella* [15,17].

Irrigation or rainfall can cause pathogens to grow in certain soils [18], likely due to increased availability of water-soluble nutrients [7]. *Salmonella* survived for 129 days in daily irrigated amended soils, 89 days longer than non-amended soils [18]. Other than irrigation, flooding events may lead to soil saturation and standing water, which may provide more favorable conditions for foodborne pathogen transmission and survival. In a field study, flooding events led to significant increases in *E. coli* levels in soils that were 0.5 and 1.5 m from the flooded area, and *E. coli* persisted in the flooded soils for over 60 days [19]. Soil or compost extracts have been utilized in the laboratory to mimic soil saturation and standing water to assess pathogen survival. In liquid extracts of soil compost, EHEC O157:H7, *Salmonella*, and *Listeria monocytogenes* exhibited an initial probable decline in numbers but showed regrowth only in the absence of indigenous microbiota [20]. *Salmonella* behavior was significantly impacted by nutrient composition in extracts of amended and unamended soils, where the length of the lag phase was significantly shorter and the maximum density was significantly higher in extracts from amended soil [12,15].

While there has been a collection of soil and water chemistry data for soil extracts containing foodborne pathogens, there has not been a thorough assessment that considers both factors over an extended period. The microbiome present in these soils and extracts is dynamic, with the levels of available carbon, phosphorus, and nitrogen likely to fluctuate due to the metabolic activities of the microbiome. The native microbes in the soil and water, serving as direct competitors for nutrients, are expected to play a role in influencing the behavior and survival of pathogens. Integrating both variables will help develop models to predict the persistence and dissemination of pathogens in flooded agricultural systems. In our research, we investigated the survival of EHEC, *Salmonella enterica*, and *Listeria monocytogenes* in two different extracts from soils commonly found in agricultural settings for the production of fresh produce. We hypothesized that pathogen survival would be enhanced with a high quantity of nutrients and inhibited by the presence of native microbes. By unraveling the dynamics of soil chemistry, microbial composition, and pathogen survival, our study establishes a groundwork for informed decision-making in agriculture. The conditions provided in this study simulate scenarios of stagnant water after intense rainfall or flooding, as well as overflow from nearby drainage that could be harboring pathogens. In detailing best practices for the management of agricultural fields’ post-flooding events for the production of fresh produce, it is necessary to have a comprehensive understanding of the factors influencing pathogen behavior.

## 2. Materials and Methods

### 2.1. Bacterial Strain Preparation

Two strains each of *Salmonella, L. monocytogenes*, and EHEC (Table 1) were used in the first phase of the study. For the second phase of the study, one representative strain was used from each species (bolded in Table 1). Both *L. monocytogenes* strains and *S. enterica* strain FSL-S10-1646 were sourced from the Food Safety Lab at Cornell University. EHEC strains were sourced from the Thomas S. Whittam STEC collection at MSU. *S. enterica* strain Mdd314 was sourced from the USDA ARS in Beltsville, MD, USA and was previously used in a soil extract study [12]. The *Salmonella* Newport strain, Mdd314, which was previously selected for resistance to 80 µg/mL rifampicin was used [15]. The *L. monocytogenes* strain, 10403S, was already resistant to 80 µg/mL streptomycin. The EHEC strain, MI-0041B, was selected for resistance to 80 µg/mL rifampicin (Thermo Scientific, Waltham, MA, USA) using the process of random mutant selection [21]. This EHEC strain was streaked onto a Luria Bertani (LB) plate (Invitrogen, Carlsbad, CA, USA) and incubated at 37 °C for 24 h. One colony was transferred to 50 mL of LB broth with 4 µg/mL rifampicin. The broth was incubated at 37 °C for 24 h with shaking. A 100 µL aliquot of this overnight culture was transferred to a new 50 mL LB broth with 40 µg/mL rifampicin and incubated at 37 °C for 24 h with shaking. A 100 µL aliquot of this overnight culture was added to 50 mL of LB broth with 80 µg/mL rifampicin and incubated for 24 h with shaking. Confirmation of growth was obtained by taking a loopful of the overnight culture and streaking onto LB media plates supplemented with 80 µg/mL rifampicin. Of the overnight culture LB broth supplemented with 80 µg/mL rifampicin, 800 µL was added to 200 µL of 75% glycerol and stored in a −80 °C freezer. All the strains were stored in Brain-heart infusion broth (BHIB) (Neogen, Lansing, MI, USA) with glycerol at −80 °C for use throughout the experiments.

For each inoculation, *L. monocytogenes* strains were streaked onto BHI plates and incubated at 37 °C. The *Salmonella* and EHEC strains were streaked onto LB plates and incubated at 37 °C. Isolated single colonies were picked and transferred into BHI broth + 80 µg/mL streptomycin for *L. monocytogenes* strains, and LB broth + 80 µg/mL rifampicin for EHEC and *Salmonella* strains. These were incubated at 37 °C without shaking for 16 h. The resultant plate count data revealed the concentration of each strain to be approximately 10^9^ CFU/mL [12].

### 2.2. Soil Sample Collection and Extract Preparation

Two different types of soil were collected in bulk from the North Dakota State University (NDSU) Beef Cattle barn and from a cornfield close to NDSU campus, which were then stored in 6–7 kg batches in large Ziploc bags at −80 °C to be used throughout the experiment, as described previously [12].

#### 2.2.1. Phase 1

The first set of experiments was conducted to evaluate the impact of the sterilization of the soil extracts on pathogen behavior. Soil extracts were prepared following the methodology developed in [12]. To prepare soil extracts, 25 g of soil was taken from the frozen sample and added to 50 mL of Milli-Q ultrapure water. These suspensions were held at 4 °C for 24 h with slow agitation at 30 rpm. After incubation, the suspensions were centrifuged at 4500 rcf; the liquid portion was kept as the soil extract and was used for the soil extract assays. A preliminary chemical analysis of the soil extracts based on total organic carbon (TOC), nitrogen, and phosphorous revealed differences in the cornfield and beef barn samples, which led them to be categorized as low-nutrient and high-nutrient, respectively (Table 2). To assess the impact of the native soil microbes on pathogen behavior, the soil extracts were divided in half and those portions were sterilized via filtration of the extract through a 0.22 µm filter. The extracts were classified as follows, high-nutrient sterile (HS), high-nutrient non-sterile (HNS), low-nutrient sterile (LS), and low-nutrient non-sterile (LNS).

#### 2.2.2. Phase 2

The second set of experiments was designed to quantify pathogen behavior in the soil extracts and assess the microbiome composition over time. Large-scale preparation of the soil extracts was made to provide sufficient volume for pathogen inoculation, chemical analysis, and DNA isolation for microbiome analysis. For each high-nutrient and low-nutrient soil, 625 g was added to 1250 mL of Milli-Q ultrapure water. Soil extract was collected the same way as described in Section 2.2.1. Aliquots of 49.5 mL were made for each soil type for pathogen experiments, and 50 mL aliquots were made for chemical analysis for each time point.

### 2.3. Inoculation of Soil Extracts

In the first phase of study, high- and low-nutrient soil extracts, which were either sterile or non-sterile, were inoculated individually with the two strains of three pathogens each. For the assay, 0.5 mL of bacterial inoculum diluted in Butterfield’s buffer (pH 7.2) was added to 4.5 mL of soil extract, for a final concentration of 10^4^ CFU/mL. The inoculated soil extracts were incubated at 15 °C for 14 d. Plate count data for each of the six strains were taken at days d0, d1, d2, d3, d4, d6, d8, d10, d12, and d14, respectively, after the time of inoculation. The samples were collected at the same time for each day. In the second phase of study, one strain from each of the three pathogen types was chosen (bolded in Table 1). Extracts were inoculated the same way as for the first phase, but only non-sterile samples were used in this phase.

### 2.4. Enumeration of Pathogens from Soil Extracts

For the enumeration of pathogens, media supplemented with antibiotics were used, as described in Section 2.1. *Salmonella* and EHEC were enumerated on xylose lysine deoxycholate (XLD) supplemented with rifampicin (XLD, BD Diagnostics, Berkshire, UK) and MacConkey agar (BD Diagnostics, Berkshire, UK) supplemented with rifampicin, respectively. *L. monocytogenes* was plated on Rapid LM (Bio-Rad Laboratories, Inc., Hercules, CA, USA) supplemented with streptomycin. XLD, and MacConkey agar plates were incubated at 37 °C and Rapid LM plates were incubated at 30 °C for 24 h. Q-Count (Color Q-Count, Spiral Biotech Inc., Norwood, MA, USA) was used for the enumeration of colonies. Data were collected at d0, d1, d4, d6, d8, d10, and d14 at the same time each day.

### 2.5. Enumeration of Mesophilic Aerobic Microbes

Plate count data for mesophilic aerobic heterotrophic microbes were collected at the same data points as described in Section 2.4. Soil extracts aliquots were diluted in PBS and plated onto LB agar and incubated at 30 °C for 24 h, similar to the process used by Shah et al. [12]. Resulting colonies were enumerated and are referred to as mesophilic aerobic microbes throughout the manuscript. To observe changes in the mesophilic aerobic microbe count in the absence of pathogens, a set of soil extracts was left uninoculated, and mesophilic aerobic microbes were sampled and quantified at the same time points.

### 2.6. Chemical Analysis of Soil Extracts

Samples of soil extracts were collected from the uninoculated non-sterile extracts for chemical analysis at the same data points as in phase 2. These soil extract samples were sent to the NDSU soil testing lab for chemical analysis. Soil extract samples were stored at −20 °C after collection and prior to the analysis. A chemical analysis was performed for total organic carbon (TOC), nitrogen (N), and phosphorus (P), as well as alkalinity, pH, and determining the concentration of sodium (Na), calcium (Ca), magnesium (Mg), potassium (K), copper (Cu), iron (Fe), manganese (Mn), zinc (Zn), and chloride ions (Cl^−^). For total N and P, Kjeldahl digestion was performed. In this method, using a selenium catalyst, an organic bound element is digested with concentrated sulfuric acid and potassium sulfate [22]. Alkalinity was measured by quantifying carbonates and bicarbonates, which can indicate the local geochemical environment. To accurately measure the quantity of carbonates and bicarbonates in a sample, a pH electrode was combined with a CO_2_ electrode [23]. For phase 1, samples of the soil extracts were taken at the start of the experiment; for phase 2, samples for the chemical analysis were taken at every sampling point as described in Section 2.3.

### 2.7. DNA Extraction

Total genomic DNA was extracted from each soil extract sample at each sample time point using the Power Soil DNA extraction kit (Qiagen, Ann Arbor, MI, USA). DNA was isolated from 3 independent replicates of each pathogen in each soil extract (HNS and LNS) as well as 3 independent replicates of each soil extract that remained uninoculated. Isolated DNA was stored at −20 °C.

### 2.8. PCR and Sequencing

The 465 bp (base pair) 16S rDNA region was amplified using PCR with V3 forward and V4 reverse primers 5′-CCTACGGGAGGCAGCAG-3′ and 5′-GGACTACHVGGGTWTCTAAT-3′, respectively [24] (Invitrogen). ThermoScientific^TM^ DreamTaq^TM^ Hot start DNA polymerase was used for the PCR. The PCR was performed using the following conditions: 2 min initial denaturation at 95 °C, denaturation at 95 °C for 40 s, annealing at 50 °C for 30 s, extension at 72 °C for 1 min, final extension at 72 °C for 7 min. The cycle of denaturation, annealing, and extension was repeated 30 times. After the final extension, the samples were held at 4 °C until they were taken out of the thermocycler, and later stored at −20 °C. The genomic region that was amplified from the samples were sent to Genomics core facility in MSU (Michigan State University, East Lansing, MI, USA) for amplicon sequencing with the Illumina MiSeq.

### 2.9. Statistical Analysis and Predictive Modelling

#### 2.9.1. Pathogen Survival

Pathogen count data from both phases of the experiment were collected for three biological and two technical replicates. CFU/mL values were log-transformed for each time point and were used for subsequent analyses. One-way analysis of variance (ANOVA) in R programming language was used to test for differences in the microbial counts and chemical composition of different soil extracts. For means that were statistically (*p* < 0.05) different, Tukey’s Honest Significant Difference (HSD) was used to separate the individual differences among the soil extracts. A generalized linear model with random effects was used to test for the effect of different nutrient levels and pathogen on the survival of the cells over different days. Estimated marginal means was used to compare the survival of different pathogens in different soil extracts (*p* < 0.05).

#### 2.9.2. 16S Sequence Analysis

The sequences that were obtained after amplicon sequencing using Illumina were analyzed using QIIME2 version 2021.4 [25]. QIIME2 was used to determine OTUs (operational taxonomical units) and their abundance. It was also used to plot a relative abundance of different taxonomical units, alpha diversity plots, and Bray–Curtis dissimilarity plots. Two types of alpha diversity plots were created; one to plot the microbial diversity in low- and high-nutrient soil extracts, and another for microbial diversity in uninoculated soil extracts, and soil extracts each inoculated with three pathogens. The plots were viewed on https://view.qiime2.org/ (accessed on 26 April 2024). The files used for viewing on this website can be accessed here (https://osf.io/2wsy8/, accessed on 26 April 2024). Similarity percentages for abundance of different taxa from days 0 to 14 was analyzed using the Vegan package in R programming language version 4.2.3. This was performed by using a modified version of the simper command [26].

#### 2.9.3. Dimensionality Reduction for Predictive Modeling

Several methodologies were explored to create a predictive model for pathogen survival rates. Initially, input data were scaled to a range between 0 and 1 by subtracting the mean from the values and subsequently dividing by the standard deviation. Non-numeric values, which represented unmeasured pathogen counts for non-inoculated control soil extract samples, were replaced with zeros. This approach was selected to retain essential data within the limited dataset, thus facilitating the inclusion of complete rows containing other measurements from these control samples in further analyses. Principal component analysis (PCA) was then utilized for dimensionality reduction using the scikit-learn PCA transformer in Python [27], retaining 95% of the variance. This approach selected the most important variables in soil chemistry and microbial composition data.

#### 2.9.4. Development and Evaluation of Predictive Models

Following data cleaning and dimensionality reduction, various modeling techniques including deep learning, machine learning, and ensemble methods were employed to develop robust predictive models. The models were trained to learn the correlation between the soil extract dataset (i.e., soil chemistry and microbial composition) and pathogen counts for day 0. Then, the models were tested using data from day 14 to assess their ability to predict pathogen survival based on the soil chemistry and microbial composition from that day. A deep learning approach involved constructing a deep neural network (DNN) with two layers and ReLU activation functions using PyTorch, a Python-based deep learning library [28]. To prevent training data overfitting, a k-fold cross-validation with three splits was used alongside an early stopping mechanism based on loss. Machine learning algorithms such as the random forest (RF) regressor and the support vector machine (SVM) regressor were also employed, using the scikit-learn library. These models were fitted using a grid search cross-validation for hyperparameter tuning to prevent overfitting. Furthermore, an ensemble model using a bagging strategy was developed to combine predictions from RF and SVM to ascertain the optimal model.

Additionally, a sequential forecasting approach using long short-term memory (LSTM) was developed to improve pathogen survival prediction. To ensure class balance for input data, data were manually split into training and testing sets so that each sequence represented a time-series data for respective soil extract samples over the data collection period. For the training of the LSTM model, initial sequential data from days 0 and 1 were used to forecast pathogen survival for subsequent days 4, 6, 8, 10, and 14. Training was performed in batches of 10 epochs until no further improvement in model validation loss was observed. This model was developed and trained using TensorFlow [29], with mean squared error (MSE) as the loss function and the Adam optimizer for the optimization of the model. Overall, the effectiveness of all models in pathogen survival prediction was assessed and compared using the MSE on the test dataset.

## 3. Results

### 3.1. Pathogen Behavior Is Influenced by Soil Extract Chemistry and Presence of Native Microbiome

**Figure 1:**
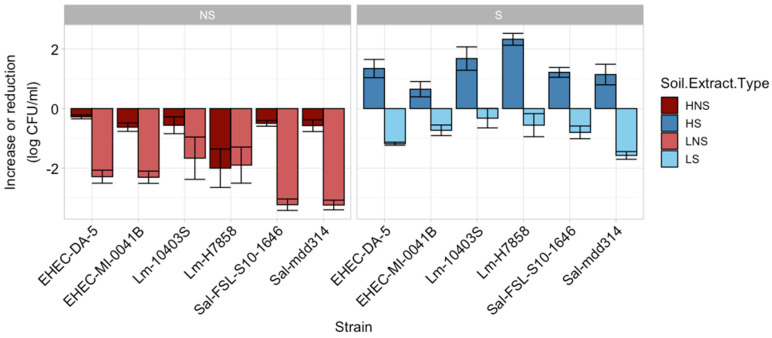
Bacterial population growth or reduction (log CFU/mL) after 14 d in high- (dark red and dark blue) and low-nutrient (light red and light blue) sterile (**right**) and non-sterile (**left**) soil extracts. Bars represent the average difference in log CFU/mL from 0 to 14 d.

For phase 1 of the study, the initial inoculum in all four soil extracts (high-nutrient sterile (HS), high-nutrient non-sterile (HNS), low-nutrient sterile (LS), and low-nutrient non-sterile (LNS)) for all strains ranged between 3.39 and 3.78 log CFU/mL (Figure S1). Over the 14-day period, strains inoculated into HS had a significant increase (*p* < 0.05) ranging from 0.65 to 2.32 log CFU/mL (Figure 1). When the native microbiome remained in the HNS soil extract, the overall pathogen reductions ranged from 0.37 to 0.62 log CFU/mL after 14 d. The low-nutrient extract did not support pathogen growth, even when sterilized. Some strains had a higher reduction, such as EHEC DA-5 (1.57 ± 0.36 log CFU/mL) and *S. enterica* mdd314 (1.18 ± 0.14 log CFU/mL), while others such as *L. monocytogenes* H7858 had an average log reduction of 0.55 ± 1.09 in the LS extract. Pathogen reductions were greatest (*p* < 0.05) in the LNS extract ranging from 1.66 (*L. monocytogenes* 10403S) to 3.24 (*S. enterica* mdd314) log CFU/mL.

Initial chemical assessment of the soil extracts showed higher levels of total nitrogen, phosphorus and carbon in the high-nutrient than the low-nutrient soil extracts (Table 2). Relative to the low-nutrient soil extract, the high-nutrient soil extract had 5× greater total nitrogen, 8× greater total phosphorus, and 9× greater total carbon.

During phase 2 of the study, there were no significant changes in chemical composition of the soil extracts over time (*p* > 0.05). We then tested for significant differences between LNS and HNS soil extracts and identified several chemical parameters that were significantly different between the extract types. Alkalinity, chloride ions, K, Na, Fe, total P, total N, and TOC were significantly (*p* < 0.05) higher in the HNS extract compared to the LNS extract.

### 3.2. Levels of Mesophilic Aerobic Microbes and Pathogens Are Affected by Soil Extract Nutrient Composition

**Figure 2:**
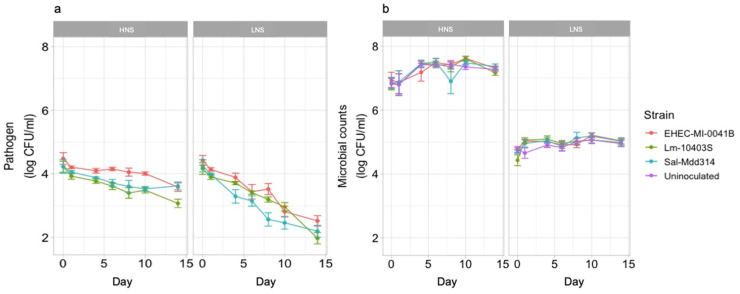
Pathogen (**a**) and mesophilic aerobic microbe (**b**) counts over 14 d in high-nutrient (HNS) and low- nutrient (LNS) soil extracts. Four colors represent the presence of different pathogens in soil extracts and an uninoculated soil extract. Each data point is an average of four biological and two technical replicates. Error bars represent the standard deviation of the counts within replicates.

Since survival was similar between strains in non-sterile extracts in the first phase, only one of each pathogen was chosen for the second phase. Pathogens had an initial inoculum of approximately 4 log CFU/mL in the HNS soil extract and declined by an average of 0.90 ± 0.73 log CFU/mL for EHEC, 1.15 ± 0.81 log CFU/mL for *L. monocytogenes*, and 0.61 ± 0.81 for *Salmonella* over 14 d (Figure 2a). Pathogen reductions were significantly greater (*p* < 0.05) in the LNS extract, with an average reduction of 1.82 ± 0.66 log CFU/mL for EHEC, 2.19 ± 0.72 log CFU/mL for *L. monocytogenes*, and 1.93 ± 0.88 for *Salmonella* over 14 d (Figure 2a). All pathogen counts were significantly different at 14 d compared to 0 d (*p* > 0.05). The average initial concentrations for native mesophilic aerobic microbes were 6.84 ± 0.l39 log CFU/mL in the HNS extract and 4.76 ± 0.28 log CFU/mL in the LNS extract (Figure 2b). Mesophilic aerobic microbes were measured for soil extracts inoculated with pathogens and those that remained uninoculated. There were no significant differences (*p* < 0.05) in mesophilic aerobic bacteria, among LNS and HNS samples that were inoculated compared to the ones that were not inoculated. Significant (*p* < 0.05) differences in the overall levels of mesophilic aerobic microbes were observed between HNS and LNS extracts, with significantly higher levels in the HNS extract (Figure 2b). At 14 d, mesophilic aerobic microbes were 7.26 ± 0.21 log CFU/mL in the HNS extract and 4.94 ± 0.27 log CFU/mL in the LNS extract.

### 3.3. Influence of Soil Extract Composition on the Microbiome

Microbiome diversity was assessed for each soil extract type over time using 16S rDNA sequencing. Overall, a greater number of taxa were identified in LNS (415) compared to HNS (370) samples. The top five taxa in HNS samples included *Pseudomonadaceae*, *Moraxellaceae, Weeksellaceae, Flavobacteriaceae,* and *Xanthomonadaceae,* while the top five taxa in LNS samples included *Burkholderiaceae, Sphingobacteriaceae, Pseudomonadaceae, Hymenobacteraceae,* and *Chitinophageceae* (Figure S2). Significant changes in relative abundance of taxa were determined between HNS and LNS soil extracts over the 14 d incubation. Taxa that were present at significantly greater levels in HNS samples included *Pseudomonadaceae*, *Moraxellaceae, Weeksellaceae,* and *Dysgonomonadaceae,* while *Flavobacteriaceae, Paludibacteraceae, Crocinitomicaceae*, and *Alteromonadaceae* were present in HNS but absent in LNS (Figure S3a) samples. Taxa that were present at significantly greater levels in LNS samples included *Sphingobacteriaceae* and *Burkholderiaceae,* unlike *Verrucamicrobiaceae, Sphingomonadaceae, Pedosphaeraceae, Enterobacteriaceae, Diplorickettsiaceae,* and *Caulobacteraceae* (Figure S3a). For HNS soil extracts, significant changes in taxa abundance over time included a decrease in *Moraxellaceae* and *Dysgonomonadaceae* from day 0 to day 14, while *Pseudomonadaceae* and *Weeksellaceae* significantly increased from day 0 to day 14. For LNS soil extracts, *Sphingobacteriaceae* and *Enterobacteriaceae* significantly increased in abundance from day 0 to day 14, while *Burkholderiaceae* and *Pseudomonadaceae* significantly decreased in abundance from day 0 to day 14.

For HNS soil extracts inoculated with EHEC, *Burkholderiaceae* and *Sphingobacteriaceae* were more abundant compared to HNS soil extracts inoculated with *Salmonella* or *L. monocytogenes* (Figure S3b). *Pseudomonadaceae*, *Moraxellaceae,* and *Weeksellaceae* were more abundant in HNS soil extracts inoculated with *Salmonella* or *L. monocytogenes* compared to HNS soil extracts inoculated with EHEC. *Moraxellaceae* decreased significantly from day 0 to day 14 for HNS soil extracts inoculated with *Salmonella* or *L. monocytogenes*, while increasing in HNS soil extracts inoculated with EHEC. For LNS soil extracts inoculated with *Salmonella*, *Weeksellaceae* and *Moraxellaceae* were present, while absent in LNS soil extracts inoculated with *L. monocytogenes* (Figure S3c). LNS soil extracts inoculated with *L. monocytogenes* had *Pedosphaeraceae* and *Bacteriovoracaceae* present, which were absent in LNS soil extracts inoculated with *Salmonella.*

### 3.4. Soil Extract Microbiome Diversity Was Not Affected by Pathogen Inoculation, but Was Influenced by Soil Extract Type

**Figure 3:**
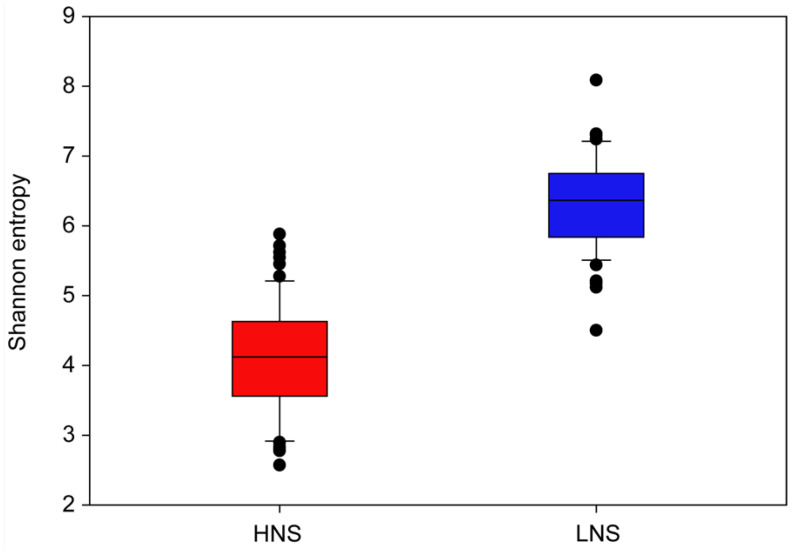
Alpha diversity (Shannon entropy) for microbiome samples based on soil extract type, viz. high- and low-nutrient non-sterile samples. Each box plot represents Shannon index values for 84 samples for each soil extract type. Boxes represent the 25th and 75th percentiles and the horizontal bar represents the median value for each distribution. Whiskers show the 10th and 90th percentiles and outliers are indicated with solid circles.

**Figure 4:**
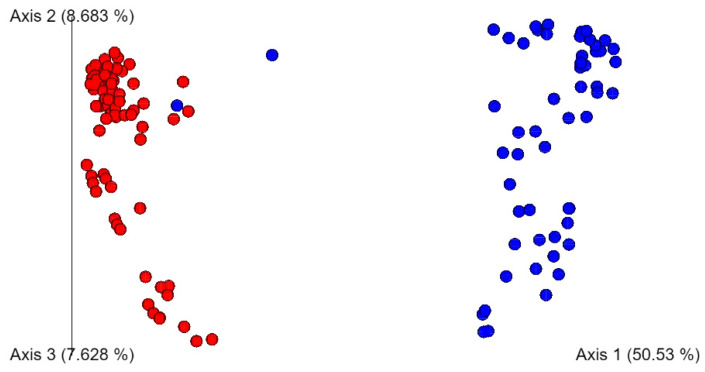
Bray–Curtis dissimilarity plot for samples from HNS (red) and LNS (blue) soil extracts.

Alpha diversity was determined for soil extracts inoculated with each pathogen, in comparison to soil extracts that remained uninoculated. The average Shannon entropy values of the soil extract microbiomes were not significantly different (*p* > 0.05) by inoculum type (Figure S4). As there were no differences in alpha diversity by inoculum type, we also examined alpha diversity by soil extract nutrient level. The average Shannon entropy for microbiomes in high-nutrient soil extracts was significantly lower (*p* < 0.05) compared to microbiomes in low-nutrient soil extracts (Figure 3), indicating greater diversity in taxa for LNS soil extracts. Beta diversity, as determined by the Bray–Curtis dissimilarity also indicated a distinct difference in soil extract microbiome composition by nutrient levels of the extracts (Figure 4).

### 3.5. Identification of Key Variables by PCA

PCA was conducted to reduce the dimensionality of the soil extract dataset, which encompassed a large number of variables related to soil chemistry and microbial compositions. The results in Figure S5 indicate that 75 principal components explained 95% of the total variance in the soil extract dataset. These components were therefore selected as the most important variables for further predictive modeling. Additionally, the PCA results showed that the observations from day 0 were more effectively represented by the major principal components compared to subsequent time points (Figure S6). It was also found that soil chemistry data exhibited a strong positive relationship with the first principal component (Figure S7).

### 3.6. Prediction of Pathogen Survival Based on Soil Chemistry and Microbial Composition

**Figure 5:**
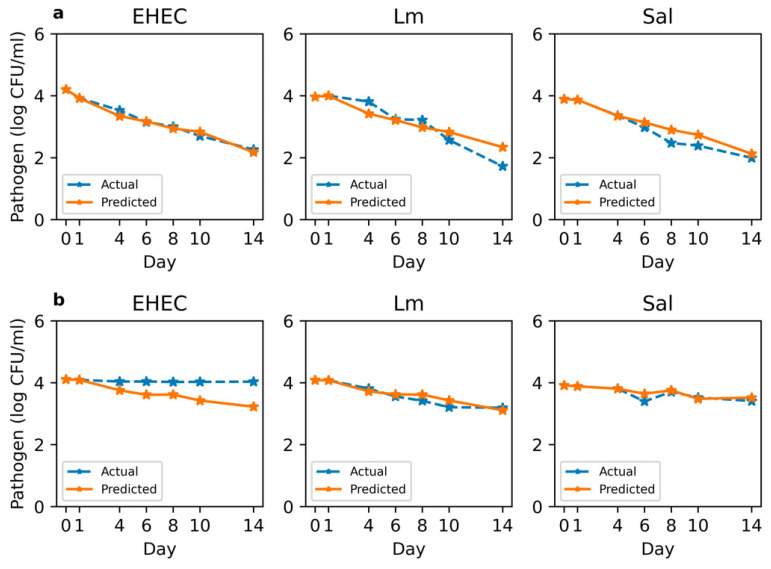
Time-series prediction of pathogen survival by the LSTM model trained on sequential datasets from days 0–1: (**a**) high-nutrient soil extract. (**b**) low-nutrient soil extract.

Using the key variables identified through PCA, various models were trained to predict pathogen survival after 14 days based on the soil characteristics. The DNN model demonstrated the least effectiveness, with an MSE of 1.545, due to the small data size that typically hinders the generalization capabilities of complex models like DNNs. Machine learning models such as RF and SVM exhibited better performance, achieving MSE values of 0.693 and 0.808, respectively. An ensemble model that integrated predictions from both RF and SVM through a bagging method slightly improved the model performance, achieving an MSE of 0.685. The LSTM model outperformed the others with an MSE of 0.09 for sequential prediction of pathogen survival after day 1. The results in Figure 5 further illustrate the variance in survival rates of each pathogen in soil extracts with different nutrient levels. Overall, the LSTM model accurately forecasted the pathogen survival trends based on the initial sequences. However, the predicted EHEC count on day 14 was lower than observed in the low-nutrient soil extract, whereas the predicted *L. monocytogenes* count was higher than observed. This performance was slightly improved when additional datasets up to day 4 were included in model training, resulting in an MSE of 0.078 (Figure S8).

## 4. Discussion

### 4.1. Significant Differences in Soil Extract Chemistry Were Associated with Differences in Pathogen Survival and Native Microbiome Diversity

Nutrient-rich soil extracts promoted pathogen growth, as shown by the greater increase in pathogen numbers in the HNS soil extracts compared to the LNS soil extracts. This is likely due to the HNS soil extracts having significantly higher amounts of TOC, P, N, Fe, Na, K, Cl^−^, and alkalinity. These findings are similar to those from other studies utilizing sterilized soil or compost extracts [30]. In sterilized soil extracts generated from poultry litter-amended and -unamended soils, *Salmonella* Newport grew over the 96 h incubation period [12]. Significantly more TOC, P, and N were found in poultry litter-amended soil extracts compared to unamended soil extracts, which was associated with a shorter lag phase and higher maximum growth levels for *Salmonella* in the amended vs. unamended soil extracts [12]. In autoclaved compost extracts with 40–50% moisture, inoculated *Salmonella* increased up to 4-log CFU/g over 3 d at approximately 24 °C [20]. Taken together, these studies indicate that the best conditions for pathogen growth are those associated with high nutrient levels in soil extracts. In soils, foodborne pathogen growth is not typically observed, though physicochemical parameters are associated with differences in survival. EHEC O157:H7 survival was positively correlated with available organic carbon and total nitrogen in soils collected from two fresh-produce-growing regions, Salinas, California and Yuma, Arizona [31]. Soil chemistry was the principal factor explaining the variation in *L. monocytogenes* survival in soils over 14 days, where the composition of Ca, Mg, and K explained 55.4% of the variability in survival profiles [13].

Other than nutrient quantity, pathogen behavior was also influenced by the presence or absence of the native microbes in the soil extract. Here, we utilized soil that was collected at a single time point and held at −80 °C until needed for the experiments. We do recognize that this storage could impact the native microbes in the soil, though any differences would be consistent throughout the experiment. Even in the presence of high nutrient levels, when native microbes were present, the pathogens were unable to increase in number. Under low-nutrient conditions, the presence of native microbes led to a greater reduction in pathogen numbers compared to when they were absent. A similar result was seen in the study utilizing poultry litter-amended and -unamended soil extracts. When the native microbes were present in the extracts, *Salmonella* Newport grew in the high-nutrient poultry litter-amended extract, though less than when it was sterilized. *Salmonella* was unable to grow in the unamended soil extracts in the presence of the native microbes [12]. Similar findings have been reported for soils treated to purposely remove native microbes. In soil microcosms, *E. coli* O157:H7 had greater survival in soils with reduced levels of native microbes than those containing the full repertoire of native microbes [32]. *E. coli* O157:H7 survival was significantly longer in autoclaved soils compared to natural soils [33]. These data all highlight the contribution of both nutrient composition and microbiomes to foodborne pathogen survival in soils and soil extracts.

The native soil microbiome is known to have antagonistic effects on the survival of foodborne pathogens including *Salmonella*, *L. monocytogenes*, and EHEC. The survival of *E. coli* O157:H7 was inversely related to microbial diversity [34,35]. Once invasion happens by a pathogen in a new environment, it can use various ways to establish in the environment including competition, antagonism, and predation. Once the pathogen invader is established in the new environment, it grows, spreads, and can displace or shift the resident taxa [36], but microbial diversity in soil can control the extent to which bacterial invaders can establish in the soil [34]. In a comparison of different soil management practices, *L. monocytogenes* was found to have steeper reductions in soils with more diversity but was not impacted by community composition [37], similar to our findings, where pathogen reductions were greater in LNS soil extracts, which had a higher level of diversity compared to HNS soil extracts.

While specific interactions between native microbiome taxa and foodborne pathogens have not been examined extensively in soils or soil extracts, some antagonistic interactions have been reported. *Pseudomonas* species, members of the family *Pseudomonadaceae,* have been shown to significantly reduce the growth of *E. coli* O157:H7 under varying environmental conditions [14]. *Pseudomonas* species have also been shown to have antagonistic effects against *Salmonella*, and *L. monocytogenes* in solutions and on the surface of fruit [38,39]. *Burkholderia* species, members of the family *Burkholderiaceae*, are known for their diversity in metabolic capabilities, including the production of numerous secondary metabolites with antimicrobial activity [40]. We observed a high abundance of *Burkholderiaceae* and *Pseudomonadaceae* in soil extracts, which could be associated with antagonistic effects toward the inoculated foodborne pathogens. We observed that *Burkholderiaceae* and *Sphingobacteriaceae* had a higher relative abundance in soil extracts inoculated with *Salmonella* or *L. monocytogenes* compared to EHEC in HNS extracts. Nutrient quantity is likely to play a role in the relative abundance of certain families in the soil extract microbiome, and some families of native microbiome can compete with certain pathogens. It is difficult to directly compare the impacts of soil and soil microbiome variables on different foodborne pathogens, as few studies have examined pathogens in the same soils or soil extracts. The data reported here constitute one of the first studies to assess multiple foodborne pathogens in the same soil extract system. More studies are needed that compare multiple pathogens in the same system.

### 4.2. AI Models Can Predict Pathogen Survival Given Key Soil Variables

Despite the challenges posed by a relatively small size of soil extract samples, effective model training results were achieved by employing PCA for dimensionality reduction (Figure 5). This strategy was crucial in preventing overfitting on the training data, given the exponential growth in sample size requirements relative to the number of input features. A commonly referenced “factor 10” rule of thumb suggests approximately 10 data points per variable, although this may be considered conservative [41]. Therefore, follow-on studies with larger sample sizes would further enhance the robustness and generalizability of our predictive modeling approach.

Among the various models tested, the LSTM model demonstrated the most effective prediction, particularly due to its suitability for forecasting applications that require an analysis of sequential data. Unlike other models, LSTM leverages memory gates to selectively retain past information and its sequential order, assisting in future predictions [42]. This attribute makes LSTM particularly useful for pattern recognition and forecasting tasks. Previous studies have also shown that LSTM models are well-suited for modeling time-series changes in biological data, including bacterial population behavior in food [43] and dynamic changes in spore concentration [44]. However, the prediction performance varied across different pathogen types and nutrient conditions (Figure 5). LSTM predictions were less accurate for EHEC in low-nutrient soil extract and *L. monocytogenes* in high-nutrient soil, but this discrepancy reflects distinct patterns in the actual survival data. Notably, our model training did not differentiate between pathogen types, thus learning a generalized trend across all pathogens. This study highlights that the interaction between soil characteristics and pathogen survival can differ among pathogen types. Future research should focus on collecting more data per pathogen type and developing pathogen-specific models, tailored to enhance accuracy and applicability in diverse environmental conditions.

## 5. Conclusions

Understanding the microbial dynamics resulting from the interplay of factors such as soil chemical composition and agricultural management practices can be instrumental in developing predictive models for pathogen persistence in agricultural systems [37]. Here, we focused on mimicking situations that could occur in agricultural fields following heavy rains and flooding events, similar to what occurred in Salinas Valley California in 2022 and January 2023 [45]. Even though pathogen numbers declined over the 14 d in both high- and low-nutrient soil extracts, *L. monocytogenes*, *Salmonella*, and EHEC persisted over this period, emphasizing the food safety concerns posed by events such as flooding and runoffs. Available macro- and micro-nutrients influenced pathogen behavior in the soil extracts, though the effects of nutrient levels were minimized by the presence of the native microbiome. These findings highlight the importance of understanding the complex interplay between soil microbiota and pathogens for effective risk mitigation. While this study explores chemistry, microbiome dynamics, and pathogen survival, future research avenues may delve deeper into the mechanisms underlying these interactions. Additionally, the implications of these findings on crop health, yield, and the broader ecosystem merit further investigation. Incorporating temporal dynamics and seasonal variations could enhance the robustness of future studies in this domain. In conclusion, this manuscript contributes significantly to our understanding of the intricate web of interactions in agricultural ecosystems. By unraveling the dynamics of soil chemistry, microbiome composition, and pathogen survival, the study paves the way for informed decision-making in agriculture, ultimately benefiting both crop production and food safety.

## Figures and Tables

**Table 1:** List of strains used. Bold letters indicate the representative strains (10403S, MI-0041B, and Mdd314) used in the second phase of study.

Strain	Species	Serogroup (Serovar)	Source
**10403S**	***Listeria monocytogenes***	**1/2a**	Skin lesion
H7858	*Listeria monocytogenes*	4b	Hot dog
**MI-0041B**	***Escherichia coli***	**O157**	Human
DA-5	*Escherichia coli*	O121	Human
FSL-S10-1646	*Salmonella enterica*	Enteritidis	Environmental, produce
**Mdd314**	***Salmonella enterica***	**Newport**	Tomato

**Table 2:** Preliminary chemical analysis results of soil extracts prepared from two different soil sources. The data are reported in parts per million (ppm) and were collected from three replicates; the average and standard deviation are reported for each compound.

Soil Extract Type	Total N (ppm)	Total P (ppm)	Total C (ppm)
High-nutrient (beef barn)	134.5 ± 25.6	22.4 ± 4.4	248.7 ± 58.8
Low-nutrient (corn field)	30.1 ± 8.5	2.8 ± 0.1	27.5 ± 2.7

## Data Availability

The original contributions presented in the study are included in the article/supplementary material, further inquiries can be directed to the corresponding author.

## Supplementary Materials

The following supporting information can be downloaded at: https://www.mdpi.com/article/10.3390/microorganisms12071498/s1. Figure S1: Survival of L. monocytogenes, EHEC, and Salmonella strains in (a) low-nutrient sterile, (b) low-nutrient non-sterile, (c) high-nutrient sterile, and (d) high-nutrient non-sterile soil extracts over a two-week period; Figure S2 Relative abundance of top 25 taxa from low- (top) and high- (bottom) nutrient soil extracts; Figure S3a: Taxa whose relative abundance differed on d0 and d14 in High and Low nutrient soil extracts; Figure S3b: Taxa whose relative abundance differed from d0 to d14; Figure S3c: Taxa whose relative abundance differed from d0 to d14; Figure S4: Alpha diversity (Shannon entropy) or microbiome samples based on inoculation with a foodborne pathogen compared to soil extract samples that remained uninoculated. Each boxplot represents the Shannon index values for 42 samples for each inoculation type. Boxes represent the 25th and 75th percentiles and the horizontal bar represents the median value for each distribution. Whiskers indicate the 10th and 90th percentiles and outliers are indicated with circles; Figure S5: Eigenvalues and explained variances of principal components (PCs) generated by PCA; Figure S6: A PCA biplot combining variables and observations across sampling time points; Figure S7: PCA loadings of top variables on selected PCs that explain significant variation in the soil chemistry and microbial composition dataset; Figure S8: Time-series prediction of pathogen survival by the LSTM model trained on sequential datasets from days 0–4.
