# Supplementary material for: Impact of Biotic and Abiotic Factors on Listeria monocytogenes, Salmonella enterica, and Enterohemorrhagic Escherichia coli in Agricultural Soil Extracts
Source: Microorganisms. 2024 Jul 22;12(7):1498. doi: 10.3390/microorganisms12071498 (PMC11278928; doi:10.3390/microorganisms12071498)
Supplement: Supplementary file 1 [file microorganisms-12-01498-s001.zip › microorganisms-3040137-supplementary.pdf]

Supplementary figures

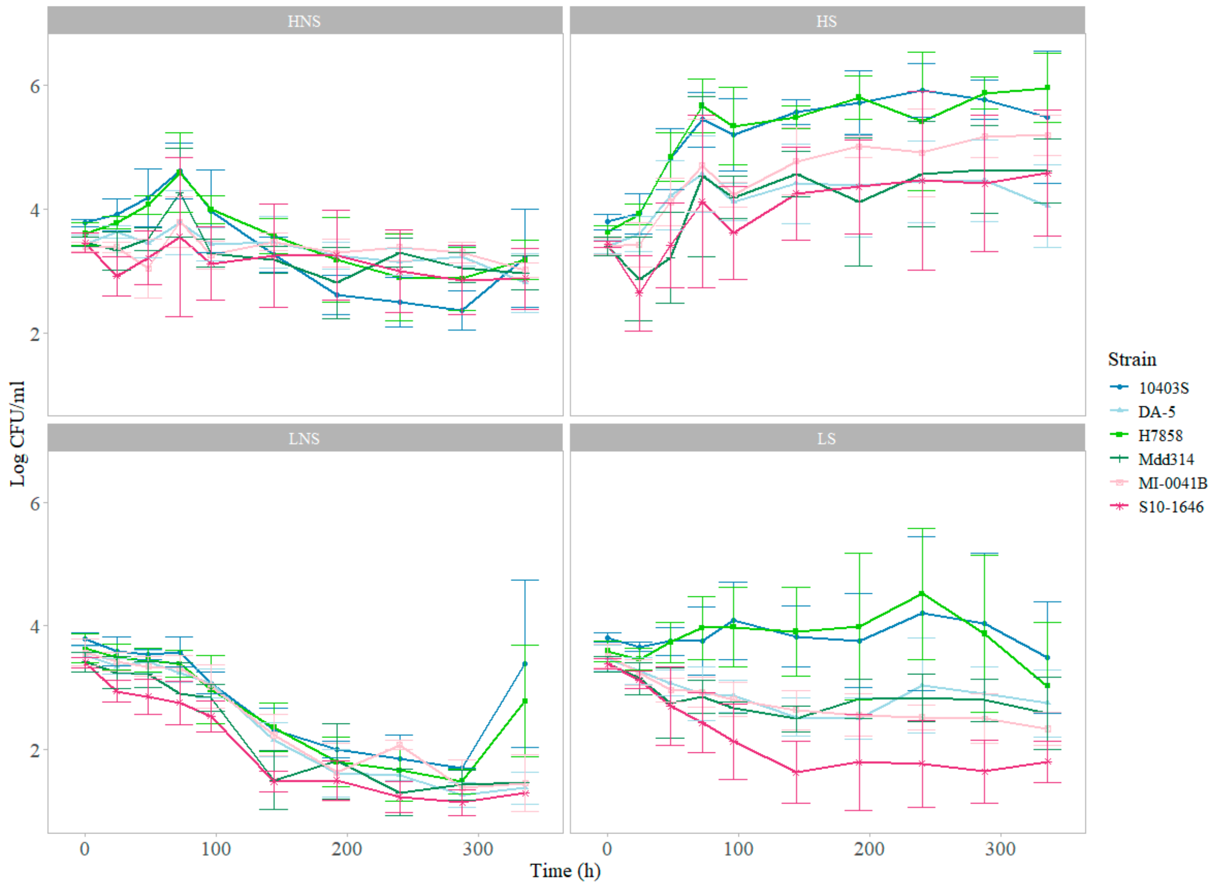

Figure S1. Survival of *L. monocytogenes* (1040S, H7858), EHEC (MI-0041B, DA-5), and *Salmonella* strains (S10 1646, Mdd314) in (a) LS-low-nutrient sterile, (b) LNS-low-nutrient non-sterile, (c) HS-high-nutrient sterile, and (d) HNS-high-nutrient non-sterile soil extracts over a two-week period. Each point represents four replicates of each strain and error bars denote SD.

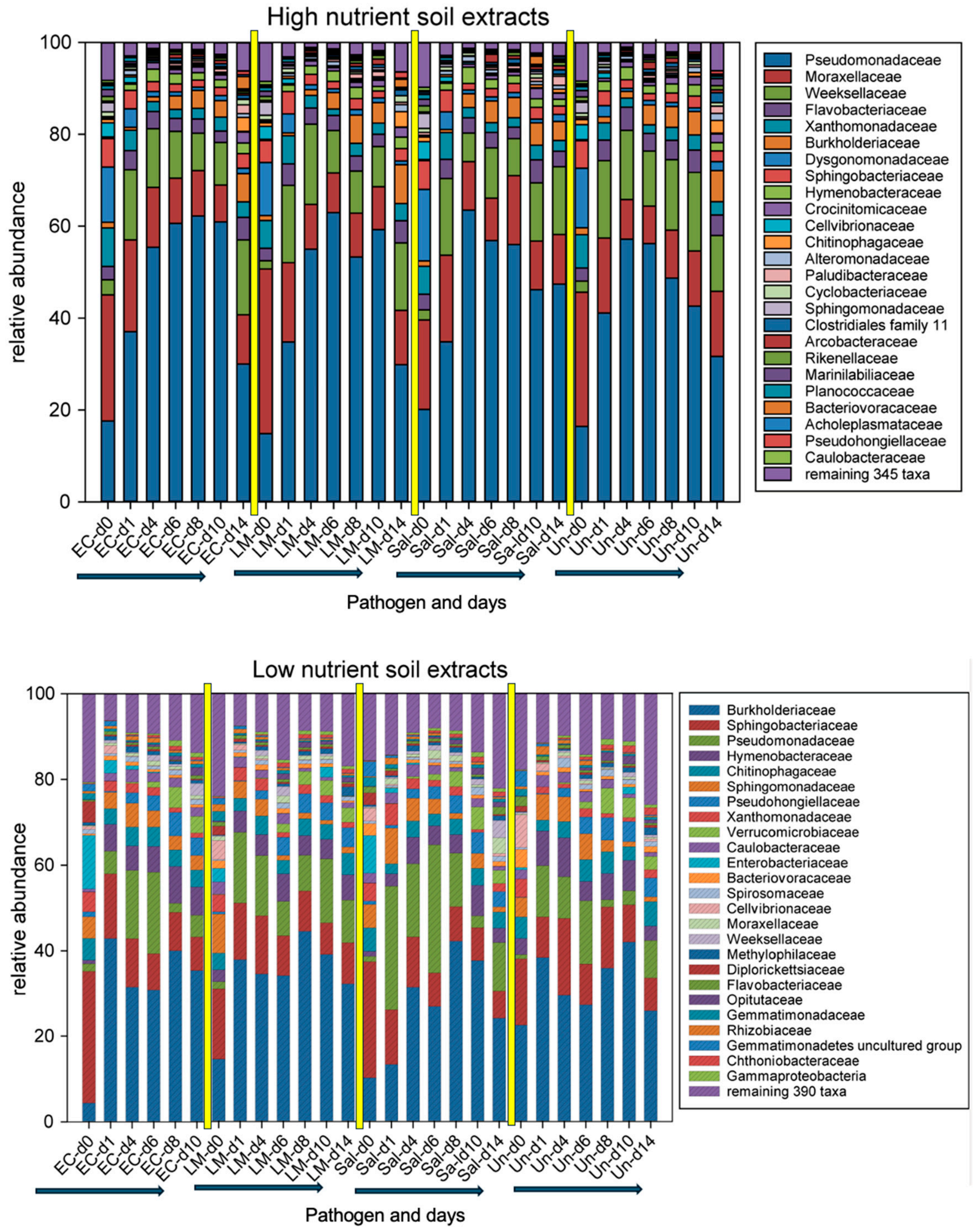

Figure S2. Relative abundance of top 25 taxa from low- (top) and high- (bottom) nutrient soil extracts. The Y-axis shows the relative abundance of bacterial families, and X-axis shows soil extracts from different days. EC, LM, Sal, and Un refer to *E. coli, L. monocytogenes, S. enterica*, and uninoculated soil extracts respectively. Days are represented by d0, d1, d4, d6, d8, d10, and d14. The black vertical bars represent separation based on soil extract type. The black horizontal arrows represent the progression of the days from 0 to 14.

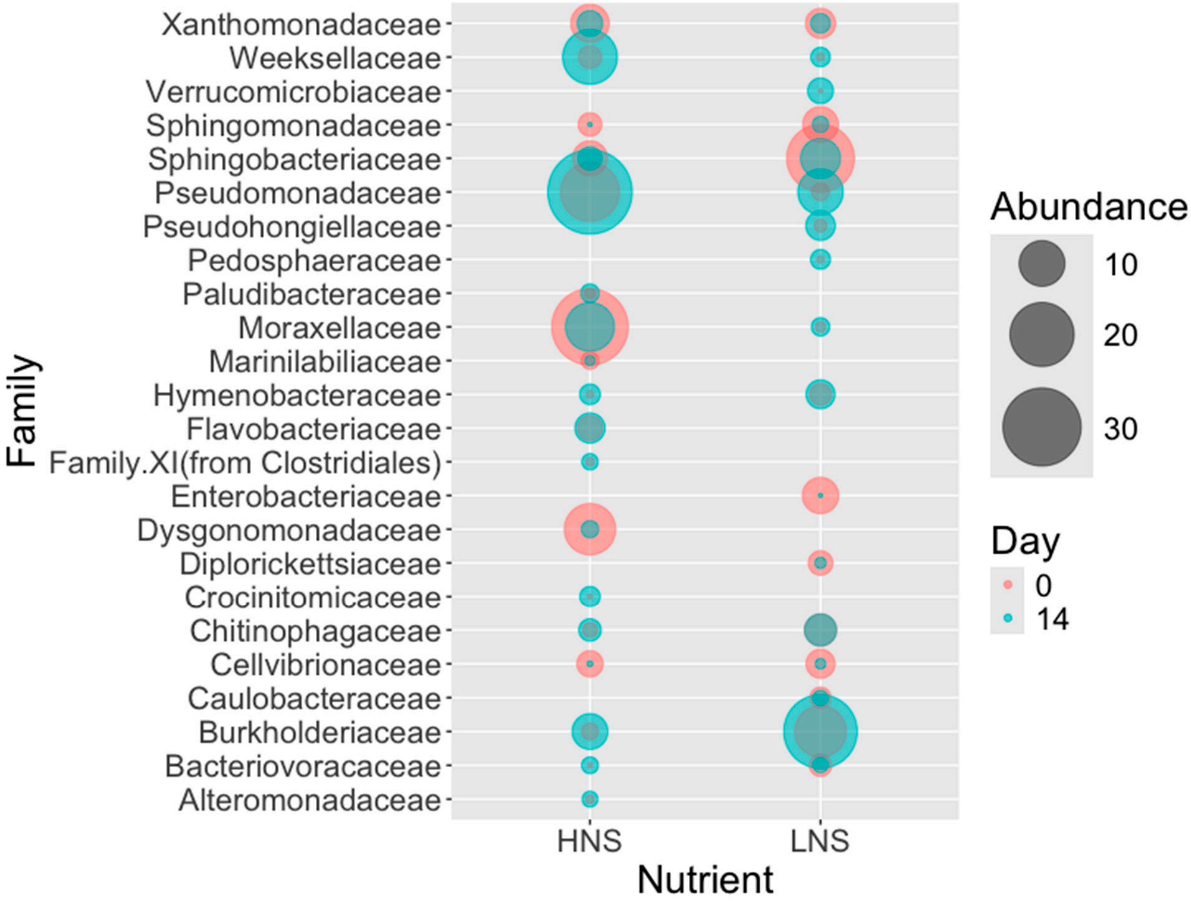

Figure S3a. Taxa whose relative abundance differed on d0 and d14 in High and Low nutrient soil extracts. The X-axis shows nutrient soil extracts. HNS and LNS represent high- and low- nutrient soil extracts respectively. The Y-axis represents taxa at Family level.

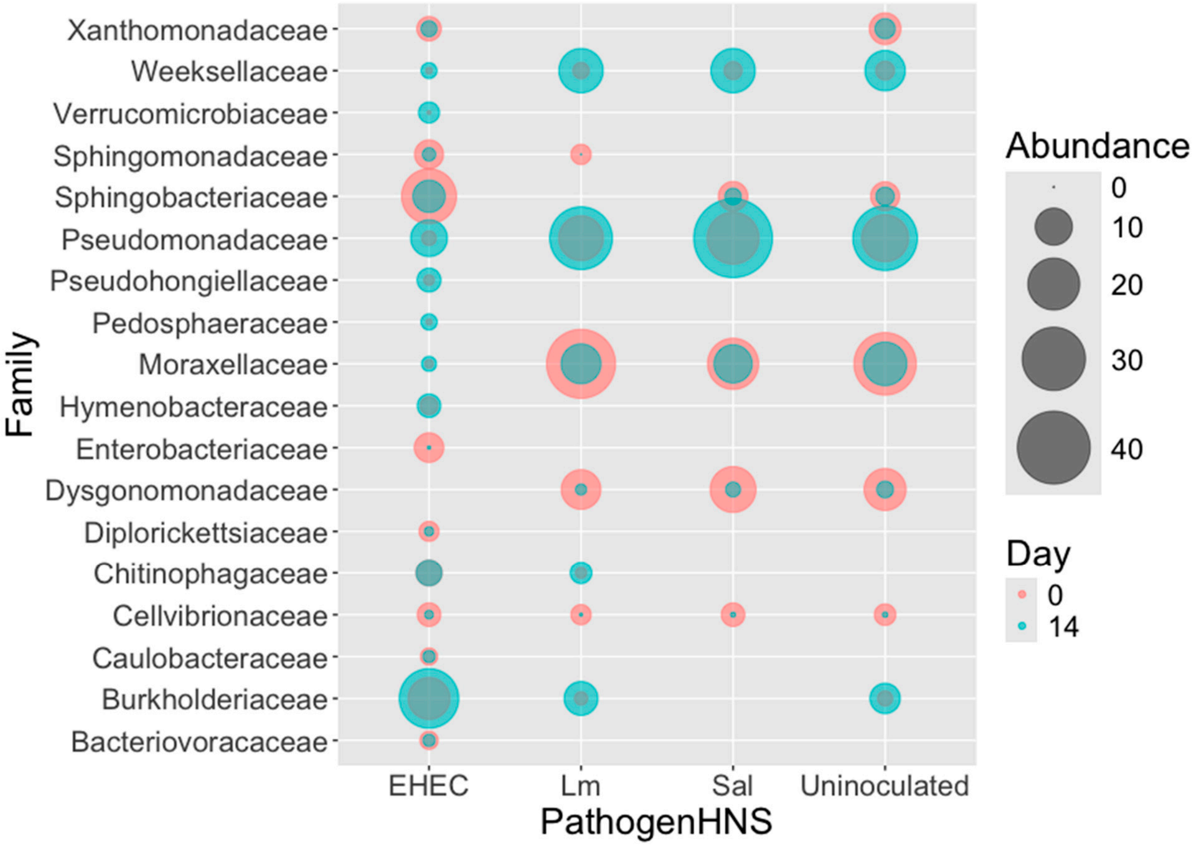

Figure S3b. Taxa whose relative abundance differed from d0 to d14. The X- axis shows bacteria in High-nutrient soil extracts; EHEC, Lm, and Sal represent soil extracts inoculated with *E. coli, L. monocytogenes, Salmonella.* and uninoculated soil extracts. The Y-axis represents taxa at Family level.

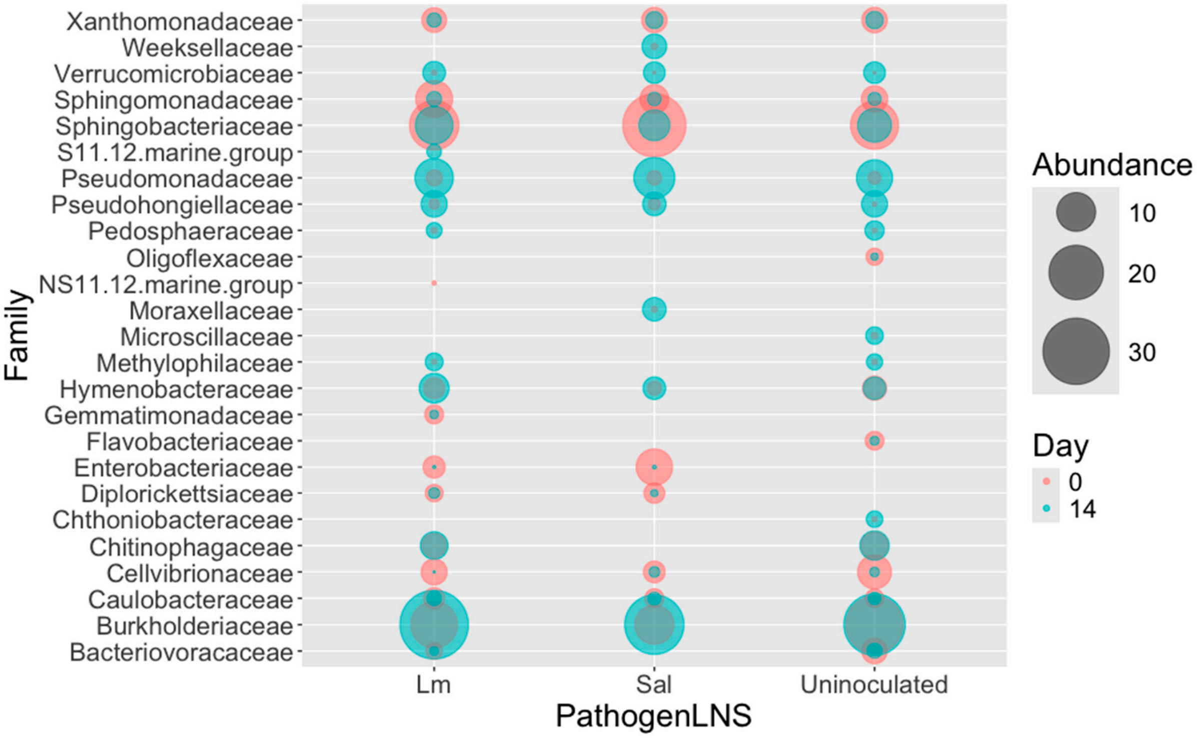

Figure S3c. Taxa whose relative abundance differed from d0 to d14. The X- axis shows bacteria in Low-nutrient soil extracts; Lm, and Sal represent soil extracts inoculated with *L. monocytogenes,* and *Salmonella.* and uninoculated soil extracts. The Y-axis represents taxa at Family level.

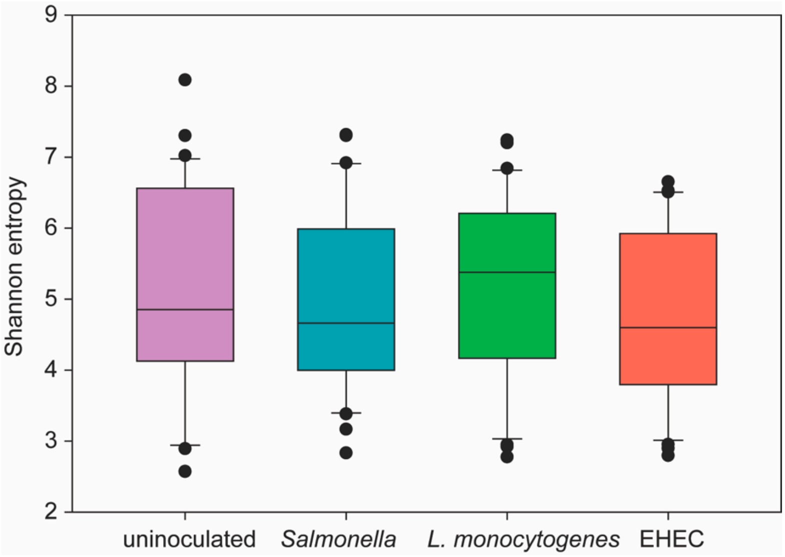

Figure S4. Alpha diversity (Shannon entropy) or microbiome samples based on inoculation with a foodborne pathogen compared to soil extract samples that remained uninoculated. Each boxplot represents the Shannon index values for 42 samples for each inoculation type. Boxes represent the 25thand 75th percentiles and the horizontal bar represents the median value for each distribution. Whiskers indicate the 10th and 90th percentiles and outliers are indicated with circles.

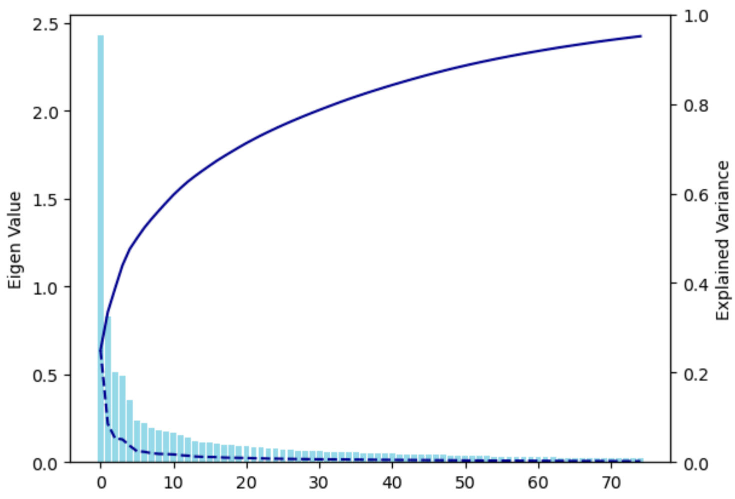

Figure S5. Eigenvalues and explained variances of principal components (PCs) generated by PCA. The dotted and solid blue lines represent the individual and cumulative proportions of the variance explained by PCs, respectively.

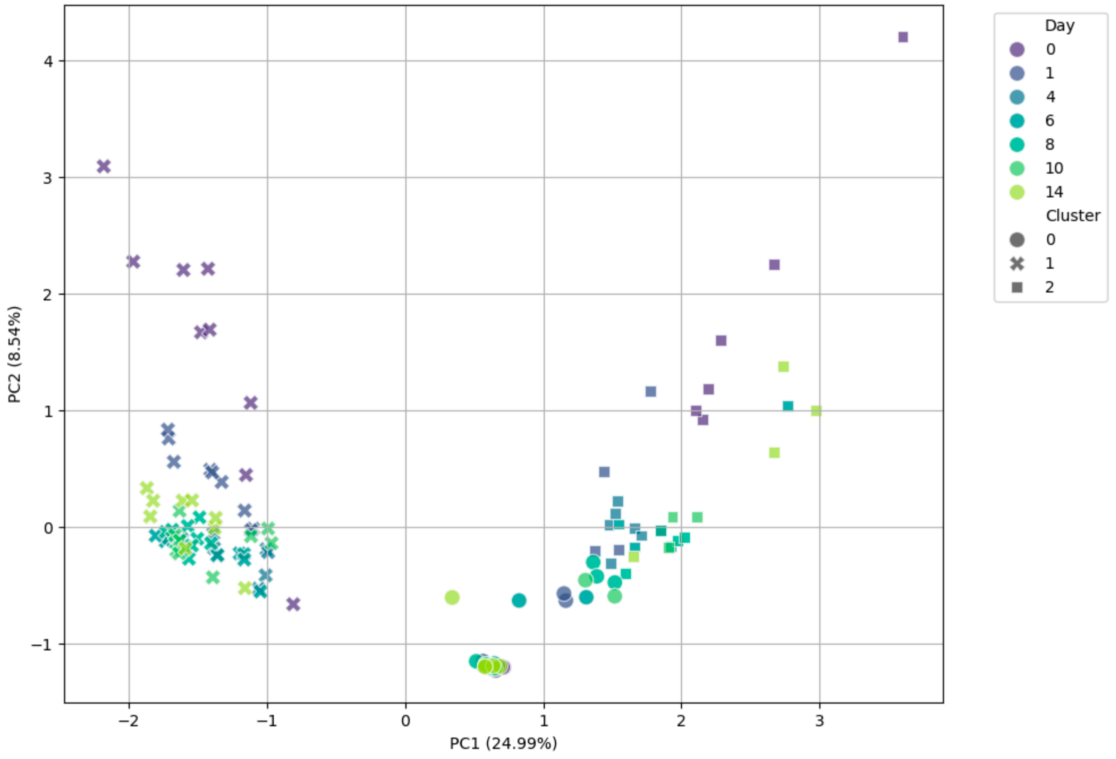

Figure S6. A PCA plot of variables and observations across sampling time points. Different clusters, identified by k-means clustering, are shown in different symbols. Their clear separation suggests that a model can effectively learn and capture patterns in the data for better predictions. PC1: the first principal component that captures the most variation in the dataset. PC2: the second principal component.

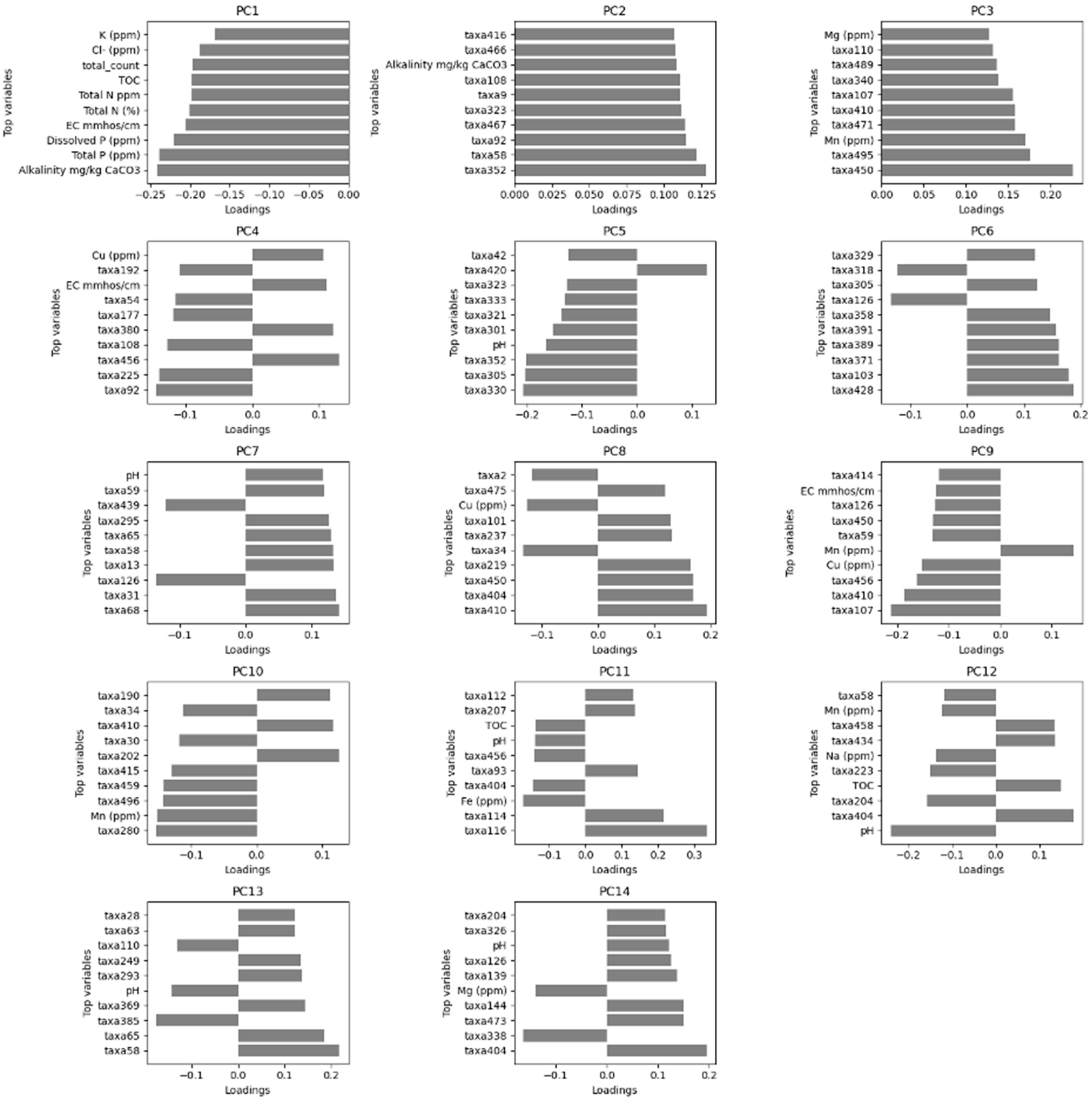

Figure S7. PCA loadings of top variables on selected PCs that explain significant variation in the soil chemistry and microbial composition dataset.

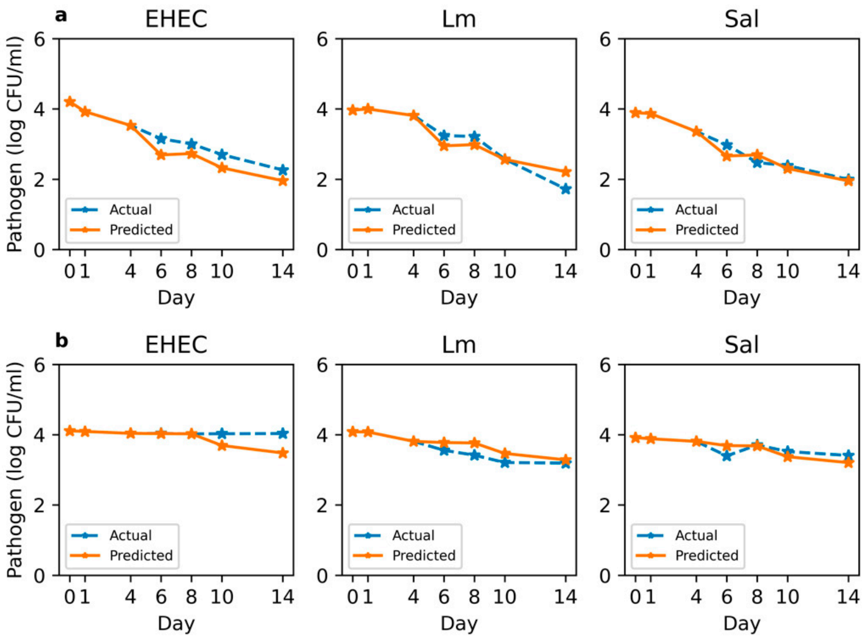

Figure S8. Time-series prediction of pathogen survival by the LSTM model trained on sequential datasets from days 0–4. Top row: high nutrient soil extract. Bottom row: low nutrient soil extract.
